# Effects of low intracellular glutathione and CbrA-CbrB-Crc signaling on methylglyoxal sensitivity in *Pseudomonas aeruginosa* LasR-deficient mutants

**DOI:** 10.1128/jb.00394-24

**Published:** 2025-09-18

**Authors:** Ana Altamirano, Marina Ruzic, Anna E. Cryan, Deborah A. Hogan

**Affiliations:** 1 Department of Microbiology and Immunology, Geisel School of Medicine at Dartmouth 12285, Hanover, New Hampshire, USA; Joseph Bondy-Denomy, University of California San Francisco, San Francisco, California, USA

**Keywords:** *Pseudomonas aeruginosa*, methylglyoxal, LasR, CbrAB, glutathione, GloA3, hydrogen peroxide

## Abstract

*Pseudomonas aeruginosa*, which causes many types of infections, often has loss-of-function mutations in the gene encoding LasR, a transcription factor involved in quorum sensing. Here, we report that LasR− strains are more sensitive to the electrophile methylglyoxal (MGO), which is produced by many cells, including those involved in immune defense. MGO, due to its high reactivity, must be rapidly detoxified to prevent the formation of damaging adducts on macromolecules. GloA3 is a GSH-dependent MGO glyoxalase involved in MGO detoxification, and we found that intracellular GSH levels were lower in ∆*lasR* strains, as measured by multiple methods. Furthermore, we show that exogenous GSH and GloA3 overexpression both enhance GloA3-dependent MGO resistance, particularly in a ∆*lasR* mutant. Previous reports suggest that one factor that contributes to the selection for LasR− strains is a growth advantage resulting from increased activity of the CbrAB two-component system, which lowers the Crc-mediated translational repression of many RNAs. Genetic data presented here suggest that low Crc activity leads to increased MGO sensitivity in LasR− strains but not through effects on GSH or GloA3. Together, these data suggest that LasR− strains are more sensitive to MGO due to multiple factors, including lower intracellular levels of GSH and decreased Crc activity, and that these factors may represent fitness trade-offs for LasR− strains.

**IMPORTANCE:**

Methylglyoxal (MGO) is a highly reactive metabolite detected in various disease states, including those involving *Pseudomonas aeruginosa*. *P. aeruginosa* requires the glutathione-dependent glyoxalase enzyme GloA3 for MGO resistance. This study reveals that *P. aeruginosa* strains with mutations in the gene encoding the transcription factor LasR, commonly found in clinical isolates, are more sensitive to MGO due to lower intracellular glutathione levels and high activity of the CbrAB-Crc regulatory pathway. Thus, sensitivity to MGO and other electrophiles may represent a trade-off for *P. aeruginosa* in infections.

## INTRODUCTION

Methylglyoxal (MGO) is a small, electrophilic, highly reactive dicarbonyl that is endogenously formed in most living cells. MGO is spontaneously produced from a variety of intermediates in sugar or glycerol metabolism, such as glyceraldehyde-3-phosphate and dihydroxyacetone phosphate (1). MGO is highly toxic because it reacts with the amines on proteins, nucleic acids, and lipids to form damaging adducts. To prevent MGO accumulation, cells have an enzymatic detoxification system called the glyoxalase system, which metabolizes MGO (1). The glyoxalase system consists of two major enzymes, glyoxalase I (Glo1) and glyoxalase II (Glo2), and is dependent on reduced glutathione (GSH) (1). Glo1 catalyzes the formation of S-D-lactoylglutathione from the spontaneously formed MGO–GSH hemithioacetal, and Glo2 converts S-D-lactoylglutathione into D-lactate as a final product (2). Other detoxification pathways in multiple organisms have also been described, such as GSH-independent glyoxalases (3) and NADPH-dependent MGO reductases (4). Elevated MGO occurs at sites of infection, cancer, diabetes, dementia, cystic fibrosis, kidney, and liver disease (1, 5–11) due to multiple factors, including increased glycolysis, which leads to an increase in pools of MGO precursors (1, 10) or reduced levels of GSH or enzymes involved in MGO catabolism (11–13). For example, cystic fibrosis (CF), a genetic disease caused by mutations in the CF transmembrane conductance regulator gene, is associated with low Glo1 and a GSH deficiency (11, 14).

*Pseudomonas aeruginosa* causes infections at multiple sites, including the respiratory tract, chronic wounds, eyes, ears, urinary tract, and blood. The success of *P. aeruginosa* as a pathogen is at least in part due to its ability to resist diverse stresses. *P. aeruginosa* has three genes encoding putative glyoxalase I enzymes, including two nickel/cobalt-dependent enzymes, GloA1 and GloA2, and the zinc-dependent GloA3 (15). The detoxification of MGO by glyoxalase enzymes occurs via covalent modification to GSH, a thiol-containing tripeptide (16). GSH is synthesized by GshA, which forms γ-glutamyl-cysteine from glutamate and cysteine, and GshB, which forms GSH through the addition of glycine (16). *P. aeruginosa gshA* and *gshB* mutants are sensitive to several different stressors, including antibiotics, bleach, hydrogen peroxide (H_2_O_2_), and MGO (17–21).

Because *P. aeruginosa* has high inherent resistance to antibiotics, particularly in biofilms, and it can become multidrug resistant, alternative antimicrobials have been sought. One such alternative is manuka honey, a unique product derived from the nectar of the *Leptospermum scoparium* plant, gathered by bees. Manuka honey exhibits potent inhibitory effects against *P. aeruginosa* growth by depolarizing and permeabilizing the membrane, which compromises the pathogen’s survival and proliferation (22). MGO is one of the major antimicrobial components in manuka honey, and both manuka honey and MGO lead to an upregulation of *gloA3* in *P. aeruginosa* (22, 23).

*P. aeruginosa* populations evolve in reproducible ways across infected individuals over time. One common type of mutation that repeatedly arises is loss-of-function mutations in the gene that encodes the transcription factor LasR, which participates in acylhomoserine-mediated quorum sensing (24). More recently, LasR has been shown to influence metabolism through the two-component system CbrAB and the downstream translational repressor Crc (25), which regulates target mRNAs involved in uptake of non-preferred carbon sources, stress response, and other metabolic processes (26–28). We previously provided strong evidence that LasR− strains have higher levels of CbrAB activity and lower levels of Crc-mediated repression of metabolism, and that these metabolic changes lead to the increased fitness of LasR− cells in passaged cultures (25). LasR− strains are commonly found in the environment and infection sites and are clinically relevant since they have been associated with worsened disease outcomes, such as in CF (29). Interestingly, LasR− mutants have been found to be sensitive to oxidative stressors such as H_2_O_2_, which was attributed to decreased expression of catalases and NADPH-producing dehydrogenases (30). The effects of MGO on LasR− strains are unknown.

In this study, we found that *Pseudomonas aeruginosa* LasR− strains are more sensitive to MGO than their LasR+ counterparts, and genetic analyses indicate that high activity of the CbrAB two-component system and low Crc repression of metabolism contribute to LasR− cell sensitivity to MGO. We show that *gloA3* and GSH biosynthetic enzymes are essential for MGO resistance and that GloA3-dependent MGO resistance can be restored by exogenous GSH. We also found that GSH levels were significantly lower in LasR− strains, compared to their LasR+ counterparts. GSH levels were not different between WT, ∆*crc*, and the ∆*crc* complemented strain, indicating that LasR− strains have increased sensitivity to MGO due to multiple independent factors, including lower intracellular GSH and lower Crc activity. These data may contribute to our understanding of trade-offs faced by the LasR− strains that frequently arise to predominance in CF lung infections but rarely persist over evolutionary time.

## RESULTS

### *P. aeruginosa* strains lacking a functional *lasR* gene have increased sensitivity to extracellular MGO

**Fig 1:**
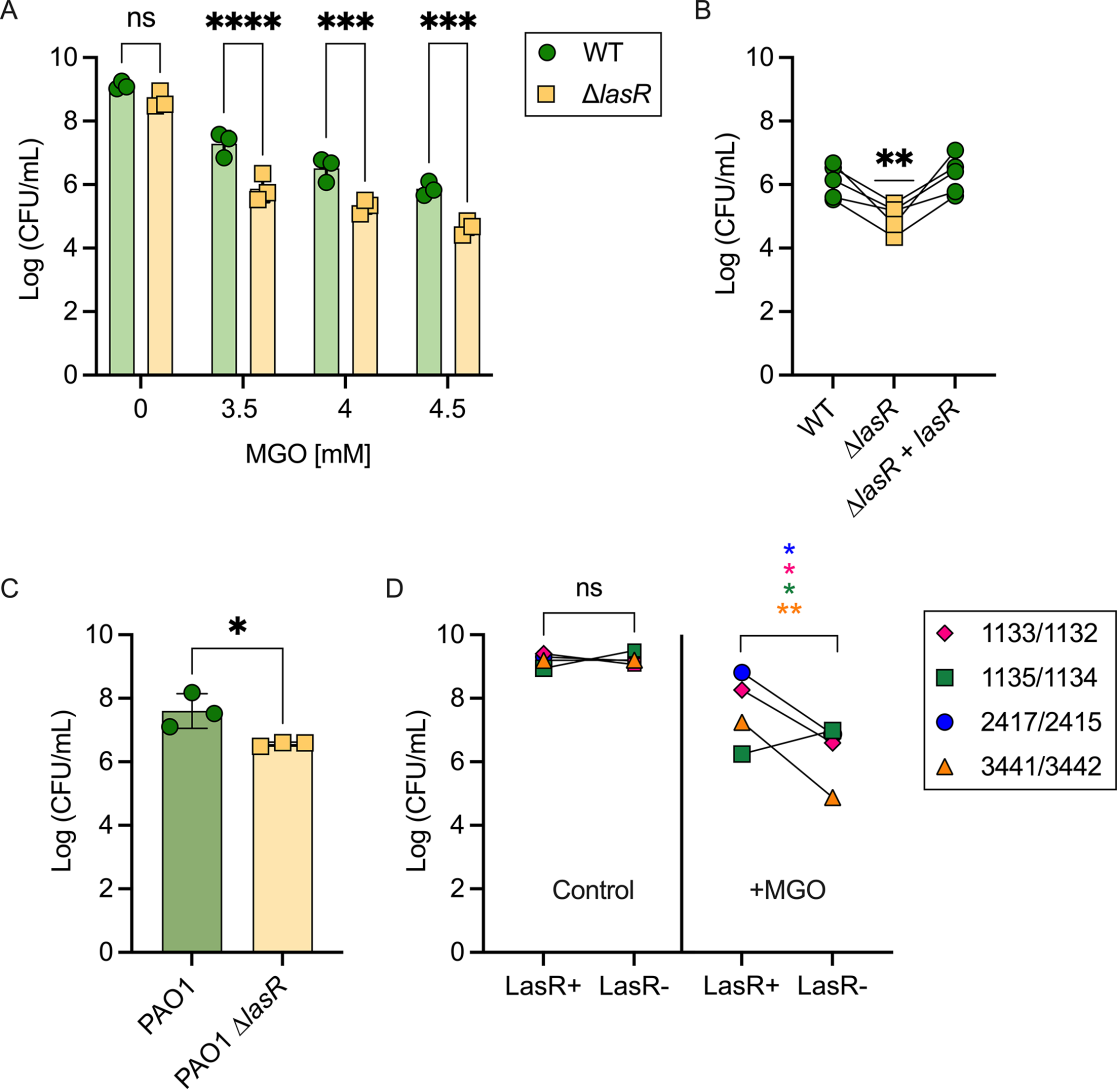
Mutations in the *lasR* gene result in sensitivity to methylglyoxal. (**A**) Methylglyoxal sensitivity assay of PA14 wild-type and *∆lasR* strains on LB agar with different concentrations of MGO. Significance between WT and ∆*lasR* was determined with a paired *t*-test (*n* = 3). (**B**) Methylglyoxal sensitivity assay at 4 mM MGO on WT, ∆*lasR*, and ∆*lasR + lasR*. A *t*-test was performed using WT mean as the theoretical mean (*n* = 5). (**C**) Methylglyoxal sensitivity assay of PAO1 WT and PAO1 *∆lasR* strains on LB agar with 4 mM MGO. Significance between WT and *∆lasR* was determined with a paired *t*-test (*n* = 3). (**D**) CFUs of four pairs of clinical isolates containing a functional and non-functional LasR, grown on LB agar and LB agar amended with 4 mM MGO. Significance was determined with a paired *t*-test. Different colors/shapes refer to different clinical isolate pairs (*P < 0.05, **P < 0.01, ***P < 0.001, and ****P < 0.0001).

 To measure the MGO sensitivity of *P. aeruginosa*, we plated PA14 WT and ∆*lasR* cells on LB containing MGO at a range of concentrations up to 4.5 mM. Between 3 and 4.5 mM, *P. aeruginosa* strain PA14 wild type (WT) had a dose-dependent decrease in colony-forming units (CFUs) (Fig. 1A). The ∆*lasR* mutant had significantly fewer CFUs on MGO-containing media when compared to the wild type across these concentrations (Fig. 1A). Based on these differences, all subsequent MGO assays were performed using a concentration of 4 mM. Complementation of the ∆*lasR* strain (∆*lasR + lasR*) restored MGO resistance levels of the WT strain (Fig. 1B). *P. aeruginosa* strain PAO1 similarly showed that the ∆*lasR* derivative was more sensitive to MGO than the wild type (Fig. 1C). All strains had similar growth when plated on media with no MGO (Fig. S1).

To further examine whether the absence of LasR activity increased MGO sensitivity, we tested multiple *P. aeruginosa* clinical isolate pairs of LasR+ (LasR functional) and LasR− (LasR non-functional) collected from the same clinical sample. The LasR+ and LasR− clinical isolate pairs had similar numbers of CFUs on LB agar. On medium with 4 mM MGO, three out of four of the LasR− clinical isolates had lower CFU counts than their LasR+ counterparts (Fig. 1D). Together, the LasR− strains were significantly more sensitive to MGO than the corresponding LasR+ strain.

### Genes involved in the CbrAB pathway play an important role in MGO detoxification

**Fig 2:**
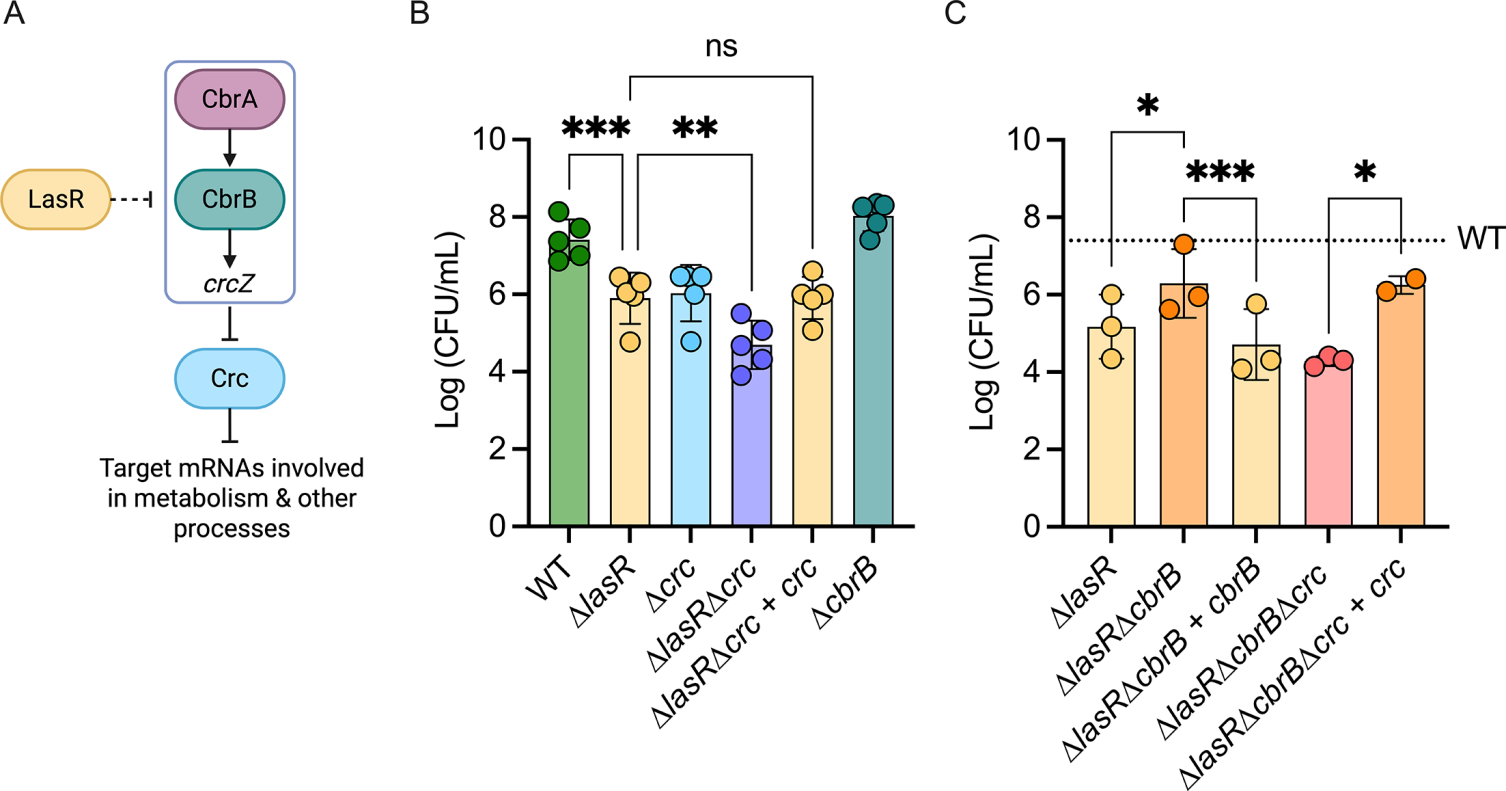
The deletion of genes in the CbrAB pathway influences sensitivity or resistance to methylglyoxal. (**A**) Carbon catabolite repression pathway: CbrAB induces expression of the small RNA *crcZ*, which then sequesters Crc to allow translation of genes involved in metabolism. Previous evidence links LasR to the repression of CbrB, but its mechanism has not been characterized. (**B and C**) Methylglyoxal sensitivity assay at 4 mM MGO of knockout strains of genes within the CbrAB pathway. Complemented strains are shown next to their knock-out counterparts. The tick line represents average WT levels for comparison. Significance was calculated using a two-way ANOVA test (*P < 0.05, **P < 0.01, ***P < 0.001, and ****P < 0.0001).

 LasR− strains have higher activity of the CbrA-CbrB two-component system (25), which leads to reduced activity of the translational repressor Crc (see Fig. 2A for pathway). Thus, we sought to determine if differences in MGO sensitivity between WT and ∆*lasR* could be, at least in part, due to differences in CbrA-CbrB-Crc pathway activity. We found that the ∆*crc* mutant had significantly worse growth on LB with 4 mM MGO than the WT, and that its sensitivity to MGO was similar to that of the ∆*lasR* mutant. Interestingly, the ∆*lasR*∆*crc* double mutant was more sensitive than the single mutants, and restoration of the *crc* gene complemented the phenotype (Fig. 2B), suggesting that LasR and Crc may partially or completely make independent contributions to MGO resistance.

In the WT and ∆*lasR* background, we also assessed the effects of deleting *cbrB*, a mutation that leads to high Crc activity (25). In the ∆*lasR* mutant, deletion of *cbrB* rendered cells significantly less sensitive to MGO. The reduced sensitivity of the ∆*lasR*∆*cbrB* on medium with MGO was reversed by complementation (∆*lasR∆cbrB + cbrB*). Consistent with the predicted pathway, the triple mutant ∆*lasR*∆*cbrB*∆*crc* but not the ∆*lasR*∆*cbrB*∆*crc* + *crc* strain had increased sensitivity to MGO relative to *∆las*R∆*cbrB* (Fig. 2C). Again, all strains had similar growth on LB medium without MGO (Fig. S1). The observed changes in MGO sensitivity upon deletion of genes encoding *cbrB* and the similar phenotypes of the ∆*lasR* and ∆*crc* strains are consistent with the model that CbrAB-Crc controlled metabolism impacts MGO resistance, and that increased activity of this pathway contributes, at least in part, to the sensitivity of LasR− strains to MGO.

### The contribution of GSH and GloA3 to protection from MGO

**Fig 3:**
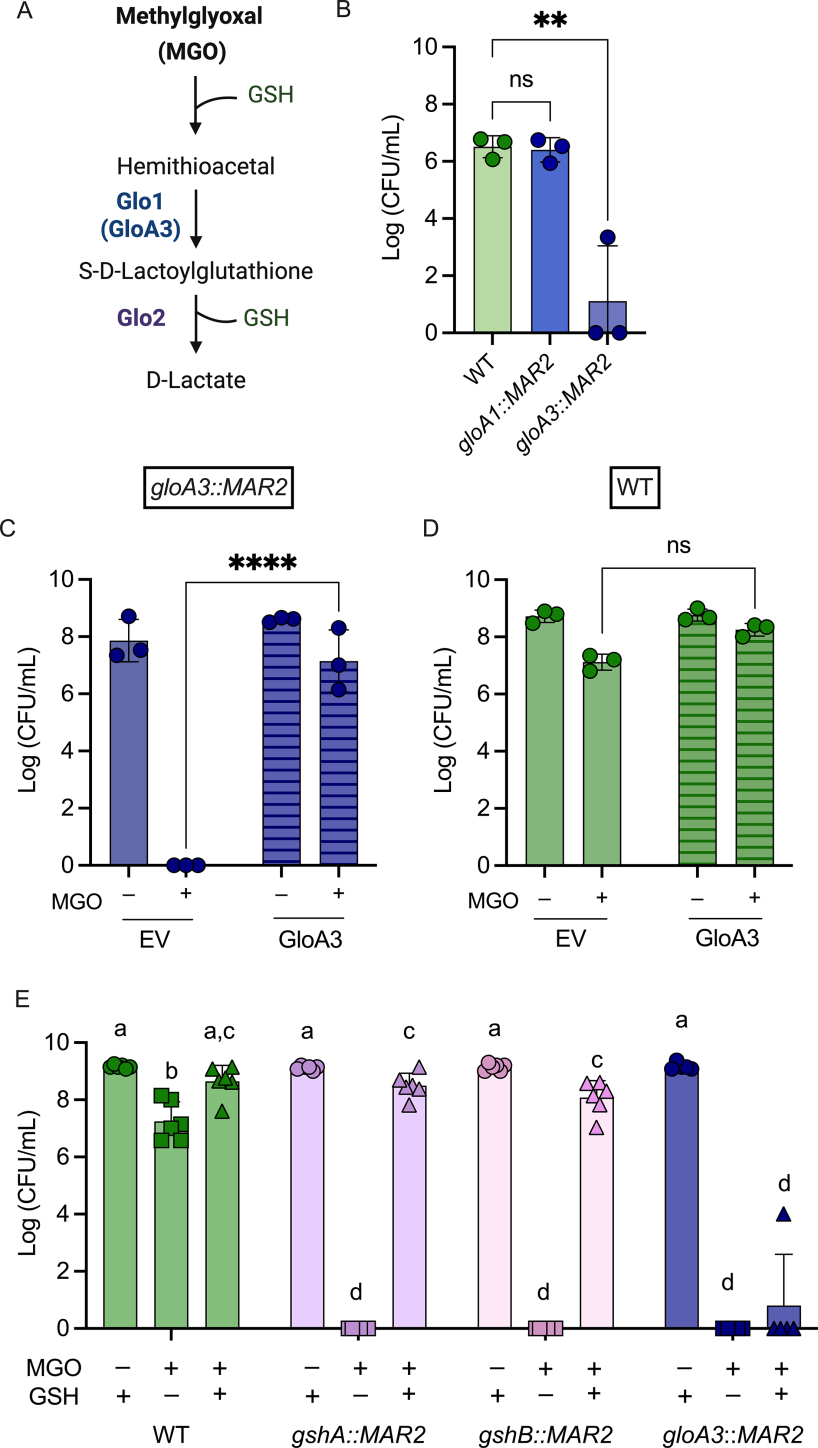
GloA3 and GSH are required for protection against methylglyoxal. (**A**) Methylglyoxal detoxification pathway by glyoxalase enzymes (Glo1 and Glo2) and reduced glutathione (GSH) to yield D-Lactate. GloA3 is a Glo1 enzyme. (**B**) Methylglyoxal sensitivity assay at 4 mM MGO of transposon mutants *gloA1*::*MAR2* and *gloA3*::*MAR2* in relation to PA14 WT (*n* = 3). Significance was calculated using a one-way ANOVA test (*P* > 0.01). (**C and D**) CFUs of (**C**) *gloA3*::*MAR2* and (**D**) WT strains containing a *gloA3* overexpression plasmid (GloA3) or an empty vector control (EV) grown in LB agar alone or amended with 4 mM MGO as indicated (*n* = 3). A two-way ANOVA was performed to look at differences between strains or conditions (**P < 0.01, ***P < 0.001, and ****P < 0.0001). (**E**) CFUs of WT and mutants lacking functional genes involved in glutathione biosynthesis (*gshA* and *gshB*) or glyoxalase 1 (*gloA3*) grown on LB agar amended with 4 mM MGO and/or 5 mM GSH as indicated. A two-way ANOVA was performed between and within media conditions. If bars are labeled with the same letter, they are not significantly different from each other (*P* < 0.01, *n* = 6).

 In *P. aeruginosa*, GloA3 is a zinc-dependent glyoxalase I enzyme that has been implicated in MGO detoxification (15) (Fig. 3A for pathway). As expected, a *gloA3*::*MAR2* transposon mutant was unable to grow on LB with MGO, while there was no significant difference between WT and a *gloA1*::*MAR2* mutant (Fig. 3B). The sensitivity of the *gloA3* mutant to MGO was rescued by constitutive expression of *gloA3* on a plasmid (p*gloA3*) but not the corresponding empty vector pMQ70_EV (pEV) (Fig. 3C). The WT + p*gloA3* strain showed a slight increase in growth on MGO compared to WT + pEV, but it was not significant (Fig. 3D). In medium without MGO, there was no significance in CFUs between either the WT or *gloA3* mutant containing either p*gloA3* or pEV (Fig. 3C and D).

GloA3 requires GSH for activity (Fig. 3A for pathway) (15), and *P. aeruginosa* strain PAO1 requires GSH biosynthetic genes *gshA* and *gshB* for MGO detoxification (21). As expected, the *gshA* and *gshB* transposon mutants in PA14 (*gshA*::*MAR2* and *gshB*::*MAR2*) were strikingly sensitive to MGO when compared to the wild type, and growth on MGO was rescued by adding GSH to the medium (Fig. 3E). In contrast, the high MGO sensitivity of the *gloA3* mutant was not rescued by exogenous GSH (Fig. 3E), indicating that GSH-mediated protection against MGO is GloA3-dependent. These results confirm that GloA3 and GSH are important for MGO resistance.

### Intracellular GSH is lower in LasR− strains

**Fig 4:**
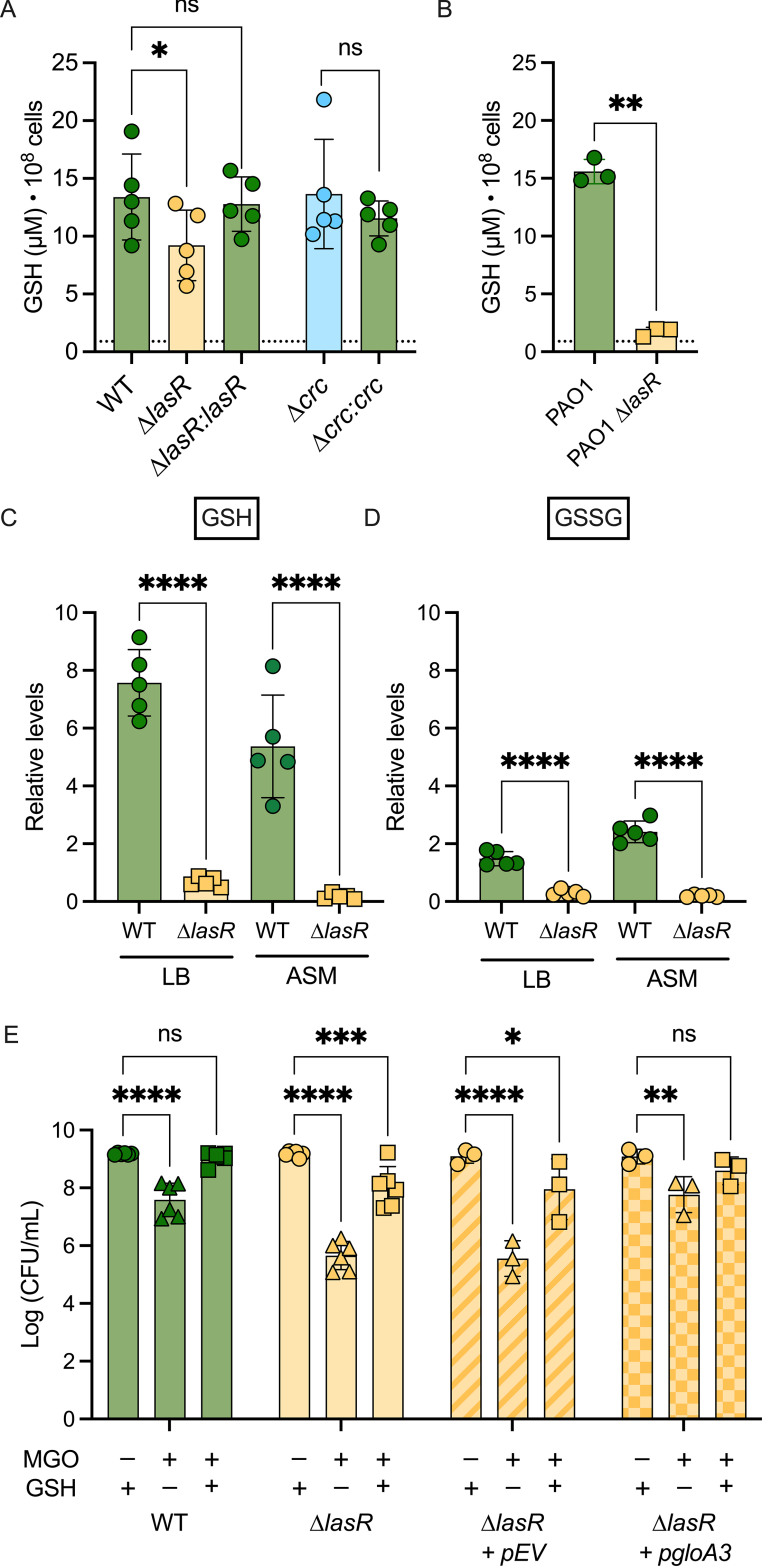
LasR− mutants have overall lower GSH levels. (**A and B**) Intracellular GSH levels of strains grown in LB media and normalized to OD = 1 (10^8^ cells), dotted line represents the *gshA*::*MAR2* mutant GSH levels; (**A**) PA14 WT, ∆*lasR*, ∆*lasR* + *lasR, ∆crc,* and *∆crc + crc* (*n* = 4), and (**B**) PAO1 WT and PAO1 ∆*lasR* (*n* = 3). Significance is determined through a paired one-way ANOVA test (*P < 0.05). (**C and D**) Relative metabolite counts of (**C**) GSH and (**D**) GSSG between WT and ∆*lasR* grown as colony biofilms in LB and ASM agar (*n* = 4) (data set from Mould et al. 2024 [33]). (**E**) CFUs of a ∆*lasR* strain containing a *gloA3* overexpression plasmid (GloA3) or an empty vector control grown in LB agar amended with 4 mM MGO and/or 5 mM GSH as indicated (*n* = 3). WT is shown for comparison. A two-way ANOVA was performed to look at differences between strains or conditions (**P < 0.01, ***P < 0.001, and ****P < 0.0001).

In light of the importance of GSH in conferring resistance to MGO, we investigated whether differences in intracellular GSH levels contribute to the varying sensitivities of WT and ∆*lasR* strains to MGO. Glutathione exists in two cellular forms: the reduced form (GSH), which is the most abundant and plays a key role in MGO detoxification, and the oxidized form (GSSG), which is typically present at much lower levels (31). We measured endogenous levels of GSH in liquid-grown WT, ∆*lasR*, and ∆*lasR + lasR* cells using a commercially available kit (see Materials and Methods for details). We found that the ∆*lasR* mutant had lower concentrations of GSH when compared to the WT and the ∆*lasR* + *lasR* complemented strain (Fig. 4A); GSH was not detected in the *gshA* mutant (indicated by a dotted line). Similarly, PAO1 wild type had significantly more GSH than the ∆*lasR* mutant (Fig. 4B). In an untargeted metabolomics analysis of strain WT and ∆*lasR* colony biofilms grown on LB agar, the levels of GSH were five times lower in the ∆*lasR* colonies compared to WT (Fig. 4C). GSSG levels were overall lower than GSH in both strains, but still lower in the ∆*lasR* mutant compared to WT (Fig. 4D). The same trend for both GSH and GSSG was seen on colony biofilms grown in artificial sputum media (ASM) (Fig. 4C and D), a medium developed to resemble the nutritional environment of the airways of people with CF (32).

Interestingly, despite the fact that the ∆*crc* mutant was more sensitive to MGO than the WT, the ∆*crc* mutant and ∆*crc* + *crc* complemented strain all had similar levels of GSH compared to the WT (Fig. 4A), and extracellular GSH was not able to rescue the MGO sensitivity of the ∆*crc* strain (Fig. S3). These data are consistent with the genetic analysis that suggested that LasR and Crc have at least some independent contributions to MGO resistance (Fig. 2). We also found that the differences in GSH did not correlate with differences in levels of *gshA* mRNA; normalized levels of *gshA* were similar between WT, ∆*lasR*, and ∆*crc* strains both in the presence and absence of MGO (Fig. S2). These data suggest that lower levels of glutathione, specifically the reduced form (GSH), may increase LasR− strain susceptibility to MGO, and that this effect is likely not mediated by Crc.

### GSH and GloA3 effects on ∆*lasR* sensitivity to MGO

To further test the model that LasR− strains are more sensitive to MGO because of lower GSH levels, we examined whether exogenous GSH could rescue the ∆*lasR* mutant sensitivity phenotype. The addition of GSH to LB medium containing MGO led to a significant increase in the growth of both the wild-type and ∆*lasR* mutant strains when compared to medium with MGO only (Fig. 4E). However, only the wild-type strain was fully rescued to CFU levels comparable to medium without MGO. Moreover, the previously published H_2_O_2_ susceptibility of LasR− strains (34) was found to be completely rescued by exogenous GSH (Fig. S4).

In addition, we assessed the effects of GSH on ∆*lasR* strains in combination with *gloA3* overexpression. We found that in the ∆*lasR* mutant, p*gloA3* led to an increase in CFUs on medium with MGO over ∆*lasR* + pEV but did not fully rescue sensitivity to medium-only CFU levels. Adding extracellular GSH further increased the number of CFUs formed on MGO-containing medium in both ∆*lasR* strains and eliminated the differences between strains with p*gloA3* and pEV (Fig. 4D). Differences in *gloA3* expression between WT and ∆*lasR* did not explain the differences in sensitivity to MGO (Fig. S2). Likewise, the ∆*crc* mutant had increased CFUs with *gloA3* overexpression on MGO-containing media (Fig. S4), and differences in *gloA3* expression did not explain sensitivity to MGO (Fig. S2). Together, these results suggest that, in ∆*lasR* strains, GSH is the limiting factor for GloA3-mediated MGO detoxification.

## DISCUSSION

**Fig 5:**
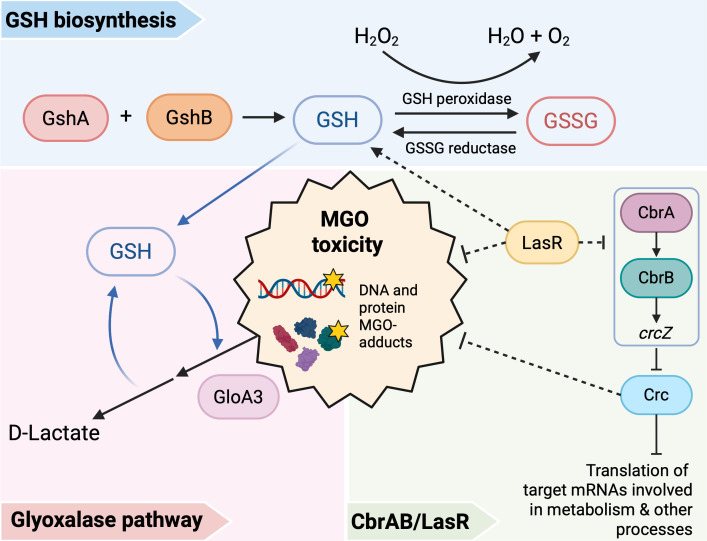
Proposed model. Factors that influence MGO toxicity in *P. aeruginosa:* (i) GshA and GshB are required for GSH biosynthesis. GSH is required for H_2_O_2_ detoxification by glutathione peroxidases, converting GSH into its oxidized form, GSSG. (ii) Glyoxalase pathway requires GSH for MGO detoxification into D-lactate. GSH is recycled in this process. (iii) LasR is required for intracellular GSH levels through an unknown mechanism. Both LasR and Crc influence resistance to MGO through unknown mechanisms.

These studies lead us to propose a model (Fig. 5) in which the high MGO sensitivity of LasR− strains is due, at least in part, to low intracellular GSH levels. Intracellular GSH concentrations were significantly lower in ∆*lasR* when compared to the wild type in both planktonic (Fig. 4A and B) and surface-associated cells (Fig. 4C) and in both PA14 and PAO1 strain backgrounds (Fig. 4A and B). Overexpression of *gloA3* alone did not fully rescue the growth differences between wild-type and ∆*lasR* strains in media containing MGO; however, increasing the availability of GSH in addition to *gloA3* overexpression eliminated these differences (Fig. 4E). Our results also indicate that the CbrA-CbrB-crcZ-Crc pathway, which is altered in LasR− mutants, contributes to MGO sensitivity, but not through changes in GSH (Fig. 2). Epistasis analyses also suggest that there are other effects of LasR loss-of-function that contribute to MGO sensitivity through unknown mechanisms (Fig. 2; Fig. S3).

The effects of MGO on microbes in infections are not well understood. MGO has antimicrobial properties, but microbes have mechanisms for MGO detoxification. One study linked neutrophil-produced MGO and *Streptococcus pyogenes* expression of an MGO-detoxifying glyoxalase I as important factors influencing outcomes in cell culture and animal model studies (35). MGO also impairs control of *Mycobacterium tuberculosis* in infected bone-marrow-derived macrophages (36). The differences in MGO sensitivity between *P. aeruginosa* among clinical isolates found in this study (Fig. 1D) are similar to previous findings in *Candida* clinical isolates. In reference 37, significant differences in MGO susceptibility among *Candida lusitaniae* isolates obtained from a single patient infected only with this species were reported. In CF *C. lusitaniae* clinical isolates, we found gain-of-function mutations in a gene encoding the transcription factor Mrr1 (38) that led to the induction of MGD1. MGD1 encodes an MGO reductase, and its increased expression led to MGO resistance and an increased capacity to use MGO as a carbon source. Interestingly, MGO induced Mrr1 regulation of other genes, including MDR1, which leads to resistance to the antifungal fluconazole and other bacterial toxins (37). Thus, MGO and changes to its transformation rate within microbial cells could influence the activity of MGO-induced pathways. In fact, MGO was shown to activate the CmrA pathway in *P. aeruginosa,* leading to the upregulation of the efflux system MexEF-OprN, which can contribute to antibiotic resistance (39).

In *P. aeruginosa*, GSH, and consequently the genes involved in GSH biosynthesis (*gshA* and *gshB*), is important for MGO detoxification (17). In this study, direct analysis of GSH levels in *P. aeruginosa* strains PA14 and PAO1 found that *lasR* mutants had a significant reduction in GSH levels relative to the wild type (Fig. 4B). These differences in GSH levels were even more pronounced in a previous metabolomics experiment using colony biofilms of PA14 wild type and ∆*lasR* (33). GSSG levels were consistently lower than GSH in all samples, and GSSG levels were higher in the wild type when compared to ∆*lasR* strains (Fig. 4C). This suggests that the observed differences in GSH levels are more likely due to differences in GSH biosynthesis or factors that affect total glutathione content, rather than differences in the ability to reduce GSSG to GSH.

The lower concentrations of GSH, a cofactor necessary for MGO detoxification by glyoxalases, may explain the increased sensitivity of LasR− strains. This likely contributes to the increased H_2_O_2_ susceptibility that has been previously documented (34), but more evidence is needed to determine the importance of GSH for protection from ROS in *lasR* mutants. In addition to detoxification pathways, it has been shown that GSH also has a role in quorum sensing by integrating information about the redox state of the cell (40), and thus, there may be a feedback loop in which the quorum sensing regulator LasR increases GSH levels. Future studies will determine if MGO formation in LasR− strains could be higher, perhaps because of increased metabolism of glucose due to high CbrA-CbrB activity (25), which would impact intracellular GSH levels (1). Currently, there is no published evidence that *P. aeruginosa* forms MGO.

Apart from MGO detoxification, GSH has multiple functions in the cell; it can protect against nitrosative and oxidative stress and even contribute to antibiotic resistance (41). Exogenous and endogenous GSH have also been shown to affect type 3 and type 6 secretion systems, swimming and swarming motility, pyocyanin production, and biofilm formation (19, 42, 43). Variations in GSH levels among different *P. aeruginosa* strains, such as in LasR− mutants, may contribute to the regulation of these systems, although further research is required to fully understand this relationship.

We previously demonstrated that metabolic adaptations play a crucial role in the evolution and success of LasR− variants (25); however, it remains important to consider how high MGO at infection sites may impact LasR− strains. The results of this study suggest that MGO sensitivity, or low intracellular GSH, could select against LasR− strains in some settings. We propose that low GSH likely represents a trade-off between metabolic and regulatory benefits associated with LasR loss-of-function that outweigh the cost of increased MGO sensitivity. Other mutations that arise may help protect LasR− strains from MGO. For example, we found one LasR− clinical isolate that was less sensitive to MGO than its LasR+ counterpart (Fig. 1D), which could be explained by mutations that lead to, for example, the upregulation of a GSH-independent MGO protection mechanism. GSH-independent glyoxalases have been characterized in *Candida albicans, Escherichia coli,* and *Staphylococcus aureus* (44–46), and recently in *P. aeruginosa* (47). Ultimately, the presence of multiple pathways for MGO detoxification likely contributes to the ability of *P. aeruginosa* strains to adapt in a wide array of environments.

## MATERIALS AND METHODS

### Strains and growth conditions

*P. aeruginosa* strains used in these studies are listed in Table S1. Each week, strains were streaked onto Luria-Bertani (LB) agar plates (10 g/L peptone, 5 g/L yeast extract, 5 g/L NaCl [LB], and 1.5% agar) from frozen stocks stored at −80°C. For overnight cultures, several colonies were transferred into 5 mL liquid LB medium in 18 × 150 mm glass tubes and incubated on a roller drum at 200 rpm for 16 h at 37°C. The pMQ70-*gloA3* plasmid, synthesized by GenScript, or the pMQ70 empty vector control was electroporated into the indicated strain. Plasmid-containing cells were maintained on carbenicillin (300 µg/mL on agar plates and 150 µg/mL in liquid LB medium) and induced with 0.2% arabinose (2 mg/mL).

### Methylglyoxal and H_2_O_2_ sensitivity assays

Overnight cultures (~16 h) were grown in LB, adjusted to an OD_600_ of 1, then serially diluted by 10-fold in 200 µL of dH_2_O in a 96-well plate (Falcon). Five microliters of each *P. aeruginosa* cell suspension dilution was spotted on the appropriate medium, dried in a biological safety cabinet for 30 min, and then incubated for 16 h at 37°C. The number of colonies from the last dilution with growth (greater than five colonies) was counted, and each plate was imaged. LB agar amended with MGO was prepared by adding the appropriate volume of MGO (purchased as 6.49 M liquid, Sigma M0252) to molten LB agar medium and then used within 3 h. The stated concentration of MGO was added from a freshly prepared filter-sterilized 0.649 M stock solution in dH_2_O. H_2_O_2_ plates were prepared similarly, using a freshly prepared, filter-sterilized 0.98 M stock (from a 30% [wt/wt] H_2_O_2_ stock) added to the molten agar to a final concentration of 300 µM, and used within 3 h. For the GSH rescue experiments, reduced L-glutathione (Sigma #G2451) was diluted in water to a final concentration of 100 mM, pH balanced (~7.4), filter-sterilized, and added to molten LB agar to a final concentration of 5 mM. Plates were used within 3 h.

### Measurement of intracellular GSH

Cells were grown in 5 mL liquid LB at 37°C on a roller drum for 16 h and then adjusted to an OD_600_ of 1.0. The GSH/GSSG ratio detection assay kit II (Abcam #ab205811) was used to measure intracellular GSH. The manufacturer’s protocol was adapted for use in *Pseudomonas aeruginosa*. In brief, normalized cells were pelleted by centrifugation and washed with 1 mL of phosphate-buffered saline. The cells were then snap-frozen and stored at −80°C for at least 24 h. Then, cells were lysed by treating with TE + lysozyme (~0.98 mg/mL) for 5 min at RT. The following steps were done on ice or at 4°C; cells and debris were removed by centrifugation at 13,000 rpm for 15 min. The supernatant was treated with trichloroacetic acid for 15 min to remove proteins from the samples. Samples were then centrifuged for 15 min at 16,000 rpm, and the supernatant was adjusted to pH = 6 by adding NaHCO_3_. Samples were centrifuged one more time for 15 min at maximum speed, and the supernatants were used in the following step. To measure GSH, eight GSH standards (0-100 µM) were prepared by diluting a 1 mM GSH stock solution (1:100). The GSH assay mixture (containing thiol green) was added to each sample and GSH standards and then incubated for 30–60 min. Fluorescence was measured at Ex/Em = 490/520 nm with a fluorescence microplate reader (BioTek Synergy Neo2). The fluorescence of the GSH standards was used to build a standard curve, and the concentration of GSH in each sample was calculated.

### Statistical analysis and figure preparation

All graphs were prepared with GraphPad Prism 10.2.0 (GraphPad Software). One-way and two-way analysis of variance tests were performed in Prism; details on each test are described in the corresponding figure legends. All *P* values were two-tailed, and *P *< 0.05 was considered to be significant for all analyses performed and are indicated with asterisks or letters in the text: *P < 0.05, **P < 0.01, ***P < 0.001, and ****P < 0.0001. Figures 2A, 3A, and 5 were created in BioRender, https://BioRender.com/nzkmd7c.

## SUPPLEMENTAL MATERIAL

Supplemental figures and table jb.00394-24-s0001.pdf Fig. S1 to S4 and Table S1.

ASM does not own the copyrights to Supplemental Material that may be linked to, or accessed through, an article. The authors have granted ASM a non-exclusive, world-wide license to publish the Supplemental Material files. Please contact the corresponding author directly for reuse.
