# Supplementary material for: Effects of low intracellular glutathione and CbrA-CbrB-Crc signaling on methylglyoxal sensitivity in Pseudomonas aeruginosa LasR-deficient mutants
Source: J Bacteriol. 2025 Sep 18;207(10):e00394-24. doi: 10.1128/jb.00394-24 (PMC12548427; doi:10.1128/jb.00394-24)
Supplement: Supplemental figures and table — Fig. S1 to S4 and Table S1. [file jb.00394-24-s0001.pdf]

Supplemental figures

A

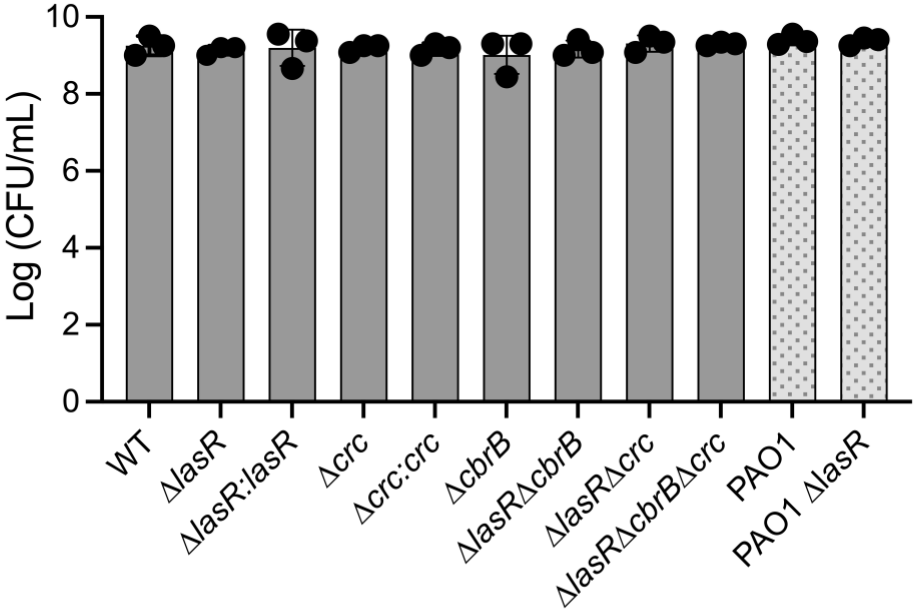

Figure S1. Growth of diverse *P. aeruginosa* strains on LB Agar. CFUs of *P. aeruginosa* PA14 and PAO1 strains normalized to OD=1 on LB agar (n=3). A one-way ANOVA was used to assess differences. All strains were non-significant from each other.

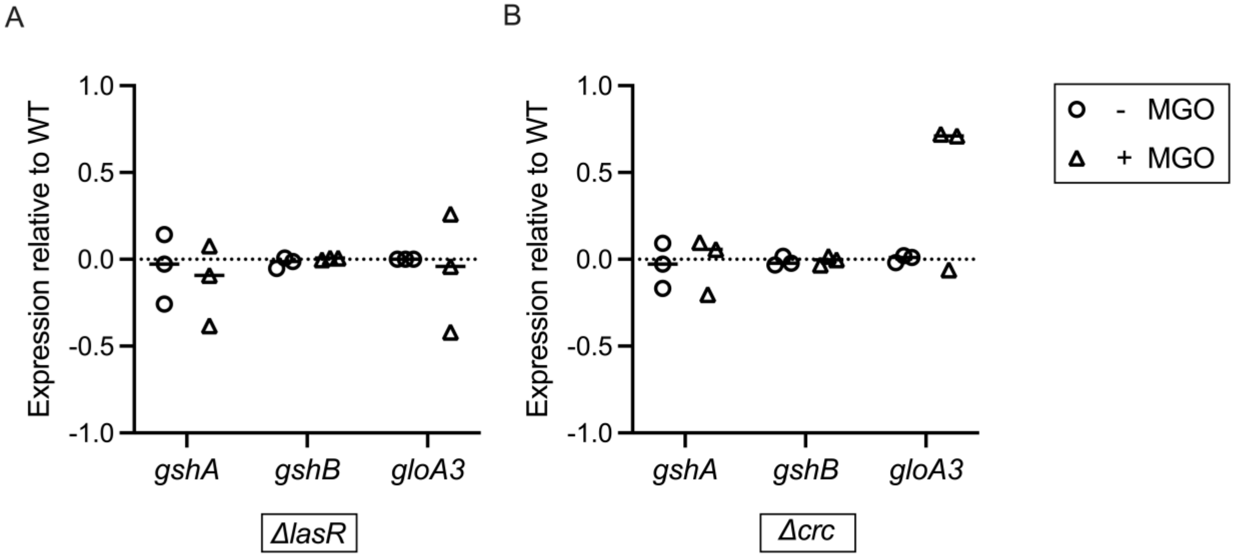

Figure S2. Stable gene expression of glutathione and glyoxalase genes among PA14 strains. Expression levels relative to WT (represented by dotted line at y=0) of genes *gshA, gshB,* and *gloA3* in (A) Δ*lasR* and (B) Δ*crc* strains grown to mid-log phase, treated with 1 mM MGO (triangle) and without MGO (circle). Expression is normalized to the housekeeping gene *rpoD* (n=3). Expression for all genes was non-significant (One-way ANOVA) when compared to the WT.

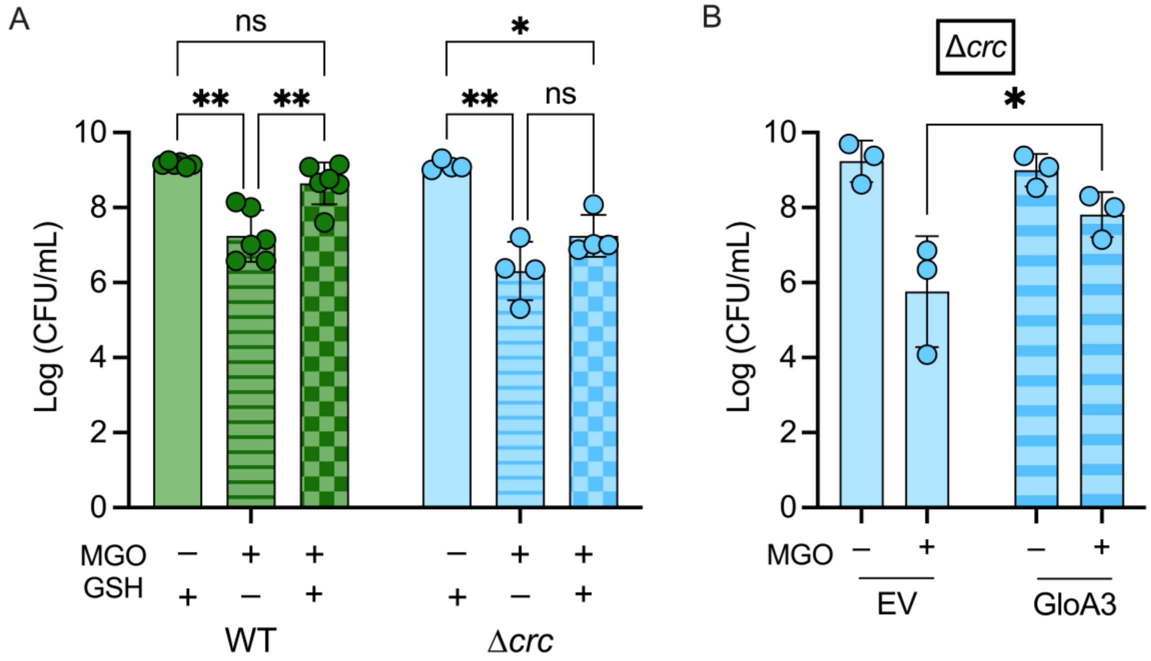

Figure S3. GloA3 overexpression, but not exogenous GSH, rescues Crc-null mutants. (A) CFUs of WT (n=6) and Δ*crc* (n=4) strains grown on LB agar amended with 4 mM MGO and/or 5 mM GSH as indicated. (B) CFUs of a Δ*crc* strain containing a *gloA3* overexpression plasmid (GloA3) or an empty vector control (EV) grown in LB agar alone or amended with 4 mM MGO as indicated (n=3). A two-way ANOVA was performed to look at differences between strains or conditions (** indicates P < 0.01, *** indicates P < 0.001, and **** indicates P < 0.0001).

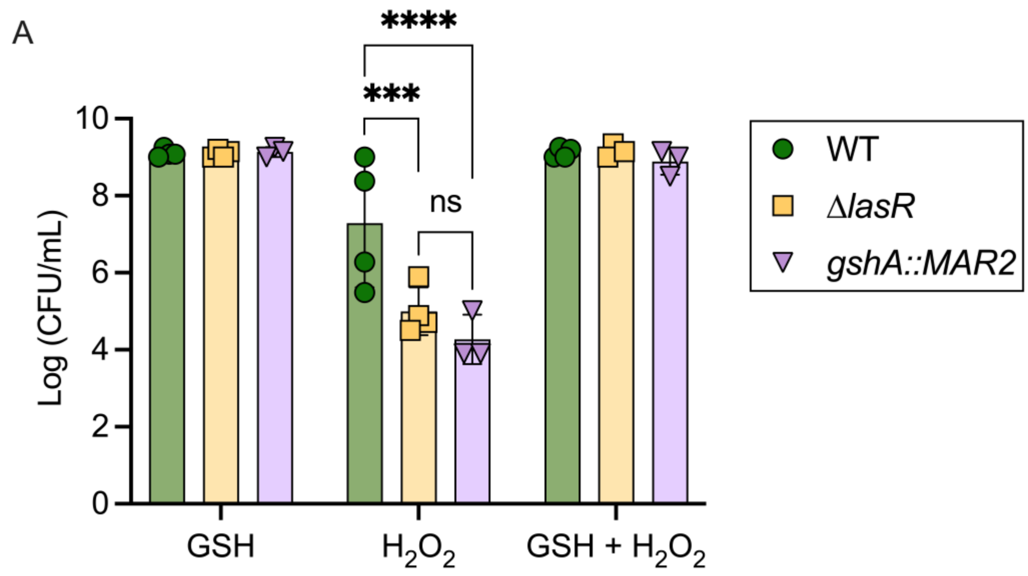

Figure S4. Extracellular GSH protects against hydrogen peroxide. CFUs of WT and Δ*lasR* (n=4), and *gshA::MAR2* (n=3) growth on LB agar media amended with 300 µM H_2O_2 and/or 5 mM GSH as indicated. A two-way ANOVA was used to measure significance. Values for strains grown in H_2O_2 are significant when compared to their corresponding GSH or GSH + H_2O_2 treatment (P<0.0001), (* indicates P<0.05, ** indicates P<0.01, *** indicates P<0.001, and **** indicates P<0.0001).

Table S1: Strains and plasmids used in this study

Strain/Plasmid	Lab ID	Description	Source
P. aeruginosa			
PA14 WT	DH123	Laboratory reference strain	(1)
PA14 Δ*lasR*	DH164	In-frame deletion of *lasR*	(2)
PA14 Δ*cbrA*	DH2507	In-frame deletion of *cbrA*	(3)
PA14 Δ*cbrB*	DH3920	In-frame deletion of *cbrB*	(4)
PA14 Δ*crc*	DH3737	In-frame deletion of *crc*	(4)
PA14 Δ*lasR*Δ*cbrB*	DH3924	In-frame deletion of *lasR* and *cbrB*	(4)
PA14 Δ*lasR*Δ*cbrB* + *cbrB*	DH3925	In-frame deletion of *lasR* and *cbrB, cbrB* complemented in native locus	(4)
PA14 Δ*lasR*Δ*cbrB*Δ*crc*	DH3926	In-frame deletion of *lasR, cbrB,* and *crc*	(4)
PA14 Δ*lasR*Δ*cbrB*Δ*crc* + *crc*	DH4186	In-frame deletion of *lasR, cbrB,* and *crc, crc* complemented in native locus	(4)
NC-AMT0101-1	DH2415	Lung CF clinical isolate with non-functional *lasR*	(5)
NC- AMT0101-2	DH2417	Lung CF clinical isolate with functional *lasR*	(5)
AMT0047-3	DH1132	Lung CF clinical isolate with non-functional *lasR*	(5)
AMT0047-2	DH1133	Lung CF clinical isolate with functional *lasR*; parent of DH1132	(5)
AMT0073-3	DH1134	Lung CF clinical isolate with non-functional *lasR*	(5)
AMT0073-2	DH1135	Lung CF clinical isolate with functional *lasR*; parent of DH1134	(5)
1260_AMT0023-30	DH3441	Lung CF clinical isolate with functional *lasR*	(6)
1261_AMT0023-34	DH3442	Lung CF clinical isolate with non-functional *lasR*	(6)
PA14 Δ*crc* + *crc*	DH3738	In-frame deletion of *crc, crc* complemented in native locus	(4)
PA14 Δ*lasR* + *lasR*	DH4740	In-frame deletion of *lasR, lasR* complemented in native locus	(4)
PA14 *gshA*::*MAR2*	DH4115	Glutamate-cysteine ligase transposon insertion mutant	(7)
PA14 *gshB*::*MAR2*	DH4116	Glutathione synthetase transposon intertion mutant	
PA14 *gloA1*::*MAR2*	DH4114	Lactoylglutathione lyase transposon insertion mutant	(7)
PA14 *gloA3*::*MAR2*	DH111	Lactoylglutathione lyase transposon insertion mutant	(7)
PA14 Δ*lasR* + pmQ70_EV	DH3804	PA14 Δ*lasR* expressing pmQ70 empty vector	(8)
PA14 Δ*lasR* + pmQ70_*gloA3*	DH4106	PA14 Δ*lasR gloA3* overexpression construct	This study
PA14 WT + pmQ70_EV	DH4103	PA14 WT expressing pmQ70 empty vector	This study

PA14 WT + pmQ70_*gloA3*	DH4102	PA14 WT *gloA3* overexpression construct	This study
PA14 Δ*crc* + pmQ70_EV	DH4108	PA14 Δ*crc* expressing pmQ70 empty vector	This study
PA14 Δ*crc* + pmQ70_*gloA3*	DH4107	PA14 Δ*crc gloA3* overexpression construct	This study
PA14 *gloA3*::*MAR2* + pmQ70_EV	DH4105	PA14 *gloA3*::Tn*M7* expressing pmQ70 empty vector	This study
PA14 *gloA3*::*MAR2* + pmQ70_*gloA3*	DH4104	PA14 *gloA3*::Tn*M7 gloA3* overexpression construct	This study
PAO1 WT	DH3537	Laboratory reference strain	(9)
PAO1-R1 Δ*lasR*	DH3538	PAO1 *lasR* deleted by gene replacement	(10)
E. coli			
S17 pmQ70_EV	DH1682	S17 expressing pmQ70 empty vector	This study
S17 pmQ70_*gloA3*	DH4109	S17 expressing pmQ70 containing *gloA3* gene	This study
**Plasmids**			
pmQ70	DH1682	Arabinose inducible expression vector, Amp^R	(11)

References:

1. Rahme LG, Stevens EJ, Wolfort SF, Shao J, Tompkins RG, Ausubel FM. 1995. Common Virulence Factors for Bacterial Pathogenicity in Plants and Animals. Science 268:1899-1902.
2. Hogan DA, Vik A, Kolter R. 2004. A *Pseudomonas aeruginosa* quorum-sensing molecule influences Candida albicans morphology. Mol Microbiol 54:1212-23.
3. Wang BX, Cady KC, Oyarce GC, Ribbeck K, Laub MT. 2021. Two-Component Signaling Systems Regulate Diverse Virulence-Associated Traits in *Pseudomonas aeruginosa*. Appl Environ Microbiol 87.
4. Mould DL, Stevanovic M, Ashare A, Schultz D, Hogan DA. 2022. Metabolic basis for the evolution of a common pathogenic *Pseudomonas aeruginosa* variant. Elife 11.
5. Smith EE, Buckley DG, Wu Z, Saenphimmachak C, Hoffman LR, D'Argenio DA, Miller SI, Ramsey BW, Speert DP, Moskowitz SM, Burns JL, Kaul R, Olson MV. 2006. Genetic adaptation by *Pseudomonas aeruginosa* to the airways of cystic fibrosis patients. Proceedings of the National Academy of Sciences 103:8487-8492.
6. Freschi L, Jeukens J, Kukavica-Ibrulj I, Boyle B, Dupont M-J, Laroche J, Larose S, Maaroufi H, Fothergill JL, Moore M, Winsor GL, Aaron SD, Barbeau J, Bell SC, Burns JL, Camara M, Cantin A, Charette SJ, Dewar K, Déziel É, Grimwood K, Hancock REW, Harrison JJ, Heeb S, Jelsbak L, Jia B, Kenna DT, Kidd TJ, Klockgether J, Lam JS, Lamont IL, Lewenza S, Loman N, Malouin F, Manos J, McArthur AG, McKeown J, Milot J, Naghra H, Nguyen D, Pereira SK, Perron GG, Pirnay J-P, Rainey PB, Rousseau S, Santos PM, Stephenson A, Taylor V, Turton JF, Waglechner N, et al. 2015. Clinical utilization of genomics data produced by the international *Pseudomonas aeruginosa* consortium. Frontiers in Microbiology Volume 6 - 2015.

7. Liberati NT, Urbach JM, Miyata S, Lee DG, Drenkard E, Wu G, Villanueva J, Wei T, Ausubel FM. 2006. An ordered, nonredundant library of *Pseudomonas aeruginosa* strain PA14 transposon insertion mutants. Proc Natl Acad Sci U S A 103:2833-8.
8. Mould DL, Botelho NJ, Hogan DA. 2020. Intraspecies Signaling between Common Variants of *Pseudomonas aeruginosa* Increases Production of Quorum-Sensing-Controlled Virulence Factors. mBio 11.
9. Holloway BW, Krishnapillai V, Morgan AF. 1979. Chromosomal genetics of *Pseudomonas*. Microbiol Rev 43:73-102.
10. Gambello MJ, Iglewski BH. 1991. Cloning and characterization of the *Pseudomonas aeruginosa lasR* gene, a transcriptional activator of elastase expression. J Bacteriol 173:3000-9.
11. Shanks RM, Caiazza NC, Hinsa SM, Toutain CM, O'Toole GA. 2006. S*accharomyces cerevisiae*-based molecular tool kit for manipulation of genes from gram-negative bacteria. Appl Environ Microbiol 72:5027-36.
